# Supplementary figures and images for: Elderly patients with cancer admitted to intensive care unit: A multicenter study in a middle-income country
Source: PLoS One. 2020 Aug 21;15(8):e0238124. doi: 10.1371/journal.pone.0238124 (PMC7442258; doi:10.1371/journal.pone.0238124)

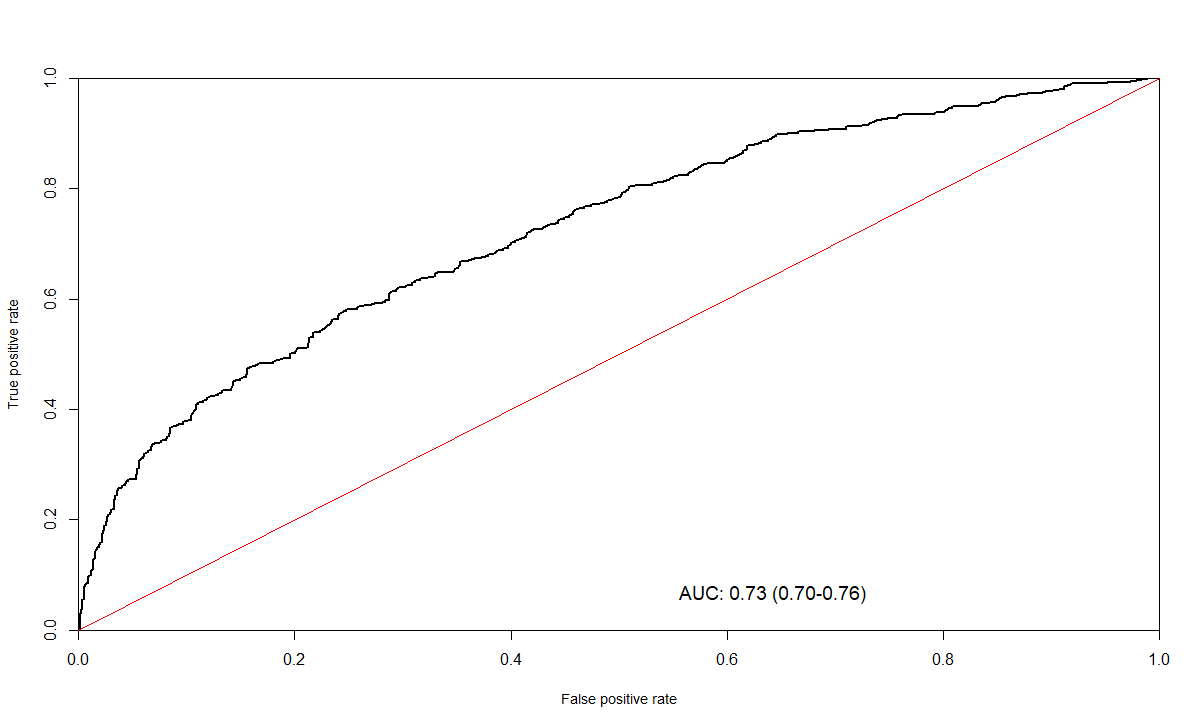


**Figure S1. Area under the receiver operator curve (AUC) for model calibration assessment**

Supplement: S1 Fig — (DOCX) [file pone.0238124.s001.docx]
