# Supplementary material for: Elderly patients with cancer admitted to intensive care unit: A multicenter study in a middle-income country
Source: PLoS One. 2020 Aug 21;15(8):e0238124. doi: 10.1371/journal.pone.0238124 (PMC7442258; doi:10.1371/journal.pone.0238124)
Supplement: S2 Fig — (DOCX) [file pone.0238124.s002.docx]

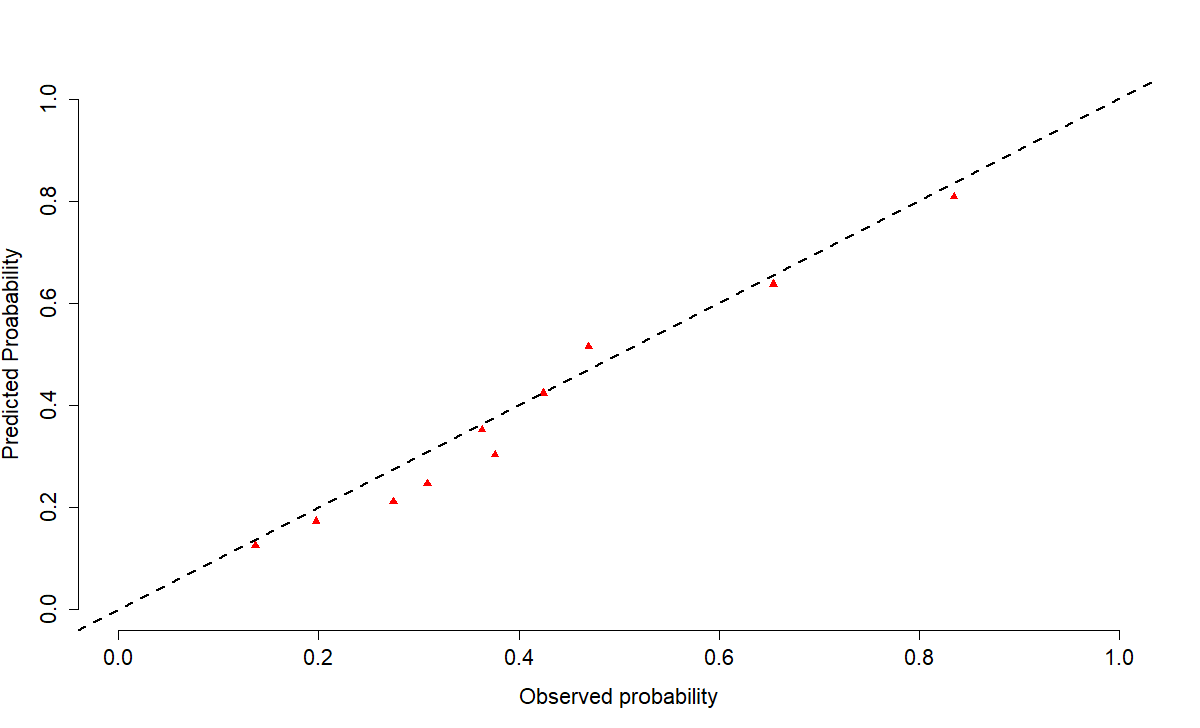


**Figure S2. Calibration curve plot**

Brier Score = 0.2021. Hosmer and Lemeshow goodness of fit (GOF) test

X-squared = 11.98, df = 8, p-value = 0.1521
