# Supplementary material for: Elderly patients with cancer admitted to intensive care unit: A multicenter study in a middle-income country
Source: PLoS One. 2020 Aug 21;15(8):e0238124. doi: 10.1371/journal.pone.0238124 (PMC7442258; doi:10.1371/journal.pone.0238124)
Supplement: S1 File — (DOCX) [file pone.0238124.s004.docx]

**Variables**

1. *Cod_ICU: Intensive Care Unit*
2. *Sex*

0 = Female

1 = Male

1. *ICU_Outcome: Status at ICU discharge*

0 = Alive

1= Dead

1. *ICU_LOS: Intensive Care Unit Length of Stay (days)*
2. *Hospital_Outcome: Status at hospital discharge*

0 = Alive

1 = Dead

1. *Hospital_LOS: Hospital Length of Stay (days)*
2. *AdmissionType: Type of admission*

1 = Medical

3 = Urgent Surgery

1. *Admission_reason: Reason for ICU admission*

1 = Urgent surgery

2 = Sepsis

3 = Cardiovascular

4 = Respiratory

5 = Neurological

6 = Renal/Metabolic

7 = Gastrintestinal

9 = Other

1. *Cancer_type = Type of cancer*

1 = Solid, locoregional

2 = Solid, metastatic

3 = Hematological

1. *Solid_site: Site of solid tumors*

0 = Hematological

1 = Bladder

2 = Anus

4 = Head and neck cancer

5 = Cervix

6 = Coloretal

9 = Esophagus

10 = Stomach

11 = Liver and biliary tree

12 = Ganglia

13 = Breast

14 = Mediastinum

15 = Sarcoma

17 = Osteosarcoma

18 = Ovary

19 = Pancreas

21 = Melanoma

22 = Non-melanoma skin

23 = Peritoneum

24 = Penis

25 = Prostate

26 = Lung

28 = Retroperitoneum

29 = Kidney

30 = Central Nervous System

32 = Thyroid

33 = Uterus

34 = Vagina

36 = Other

37 = Small intestine

1. *Hematological_type: Type of hematological cancer*

1 = Multiple myeloma

2 = Leukemia

3 = Lymphoma

99 = Other

1. *MV = Mechanical ventilation*

0 = No

1 = Yes

1. *Vasopressors*

0 = No

1 = Yes

1. RRT: Renal replacement therapy

0 = No

1 = Yes

1. *Charlson: Charlosn Comorbidity Index (points)*
2. *SAPS3: Simplified Acute Physiology Score 3 (points)*
3. *PS: Performance Status*

0 = ECOG 0-1

1 = ECOG 2-4

*ECOG = Eastern Cooperative Oncology Group
